# Gorham-Stout syndrome: the radiologic-pathologic correlation as a diagnostic pathway when bone is vanishing

**DOI:** 10.1590/0100-3984.2017.0145

**Published:** 2019

**Authors:** Rubens Gabriel Feijó Andrade, Ana Paula Vieira Fernandes Benites Sperb, Bruno Hochhegger, Osvaldo Andre Serafini, Simone Gianella Valduga

**Affiliations:** 1 Hospital São Lucas - Pontifícia Universidade Católica do Rio Grande do Sul (PUCRS), Porto Alegre, RS, Brazil.; 2 Centro Clínico - Pontifícia Universidade Católica do Rio Grande do Sul (PUCRS), Porto Alegre, RS, Brazil.; 3 Universidade Federal de Ciências da Saúde de Porto Alegre (UFCSPA), Porto Alegre, RS, Brazil.

*Dear Editor,*

**Figure 1:**
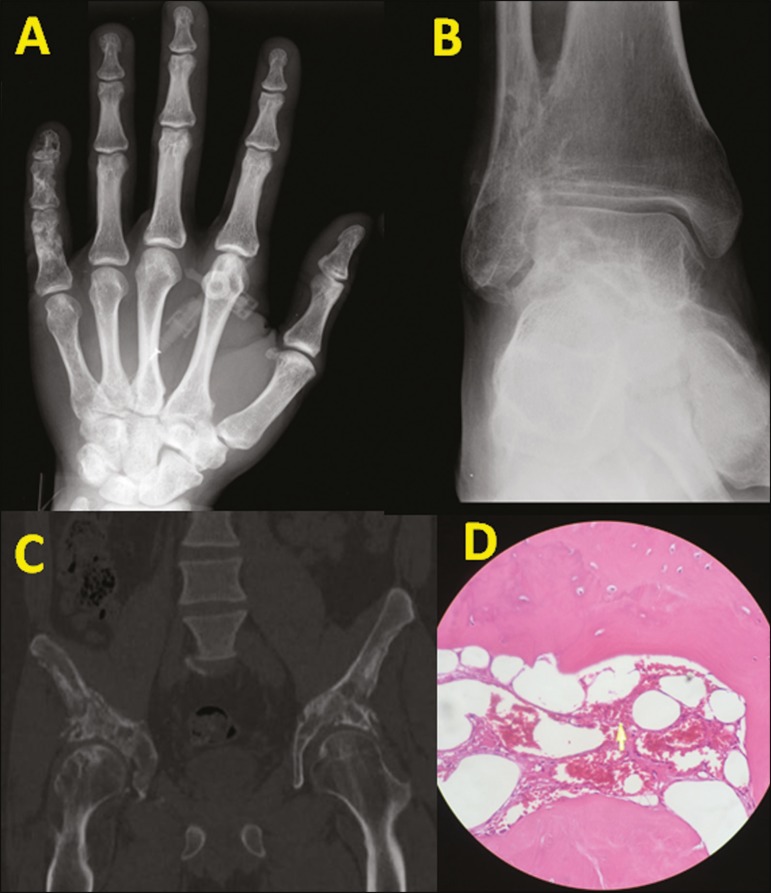
**A:** X-ray of the right hand, demonstrating permeative osteolytic lesions in the middle and distal phalanges of the fifth finger. A similar pattern is seen on computed tomography: lesions of the left malleolus and fibular metadiaphysis (**B**); and lesions affecting the iliac and femur (**C**). **D:** Histology of the lesion biopsied in the right iliac, showing cavernous proliferation of intraosseous capillaries throughout the preserved bone tissue (hematoxylin-eosin staining).

A 34-year-old previously healthy man presented with a 12-month history of progressive polyarthralgia and edema of the hips, right ankle, and intercostal spaces. He reported no history of trauma. Conventional radiography revealed several mixed lesions (predominantly osteolytic lesions) in the pelvic ring, proximal femur, distal femur, distal tibia, both tali, and lumbar vertebral bodies, as well as unconsolidated fractures of the costal arches, with no periosteal reaction or associated soft tissue changes (Figure 1). The initial hypotheses of multifocal osteolysis were secondary osteolytic conditions such as infection, cancer (primary or metastatic), inflammatory disorders, and endocrine disorders. The results of laboratory tests (complete blood count, protein profiles, parathyroid hormone level, ionic calcium level, and phosphate level) were normal, as were those of computed tomography staging. After the primary hypothesis of multifocal osteolysis had been excluded, the radiological hypothesis of Gorham-Stout syndrome was proposed. The patient was submitted to surgical biopsy of the right iliac bone, and the histopathology demonstrated spongy bone marrow tissue, the bone marrow having been replaced by fibrovascular tissue with innumerable capillary and cavernous vessels, cortical bone resorption, and reactive immature bone, consistent with massive osteolysis (Figure 1).

Initially described as "missing bone disease with intraosseous vascular alterations"^(^^1^^,^^2^^)^, the monocentric form of osteolysis of a bone or contiguous area of bone, with a predilection for the axial skeleton, was subsequently dubbed Gorham-Stout syndrome. The process is usually monostotic, although there have been a few reports of cases in which it was polyostotic^(^^3^^)^. In decreasing order of frequency, it affects the scapula, proximal end of the humerus, femur, rib, iliac bone, ischium, and sacrum^(^^3^^)^. It is currently known by a variety of names, including massive osteolysis, idiopathic osteolysis, vanishing bone disease, disappearing bone disease, phantom bone disease, spontaneous bone absorption, progressive bone atrophy, bone hemangiomatosis, and lymphangiomatosis of bone.

Gorham-Stout syndrome primarily affects children and young adults, the affected individuals presenting nonspecific complaints such as pain and joint edema. The pathological mechanisms proposed to date are multifactorial, with no known triggering factor. The histopathological hallmark is the replacement of normal bone by fibrous tissue with aggressive, expansile, non-neoplastic proliferation of capillary or cavernous blood vessels^(^^3^^-^^7^^)^, a process that can culminate in the replacement of the entire bone by fibrous tissue^(^^7^^)^. Histopathologically and radiologically, the syndrome initially presents as foci of rarefaction in the bone marrow and subcortical bone, with slow, irregular progression that can result in effacement of the diaphysis of the bones, narrowing of the involved ends, and, in some cases, the complete disappearance of the bone. Pathological fractures that do not consolidate are common and are characteristic of the syndrome^(^^1^^,^^8^^)^. The pathological-radiological diagnosis proposed by Heffez et al.^(^^6^^)^ is based on specific criteria, including positive biopsy findings for angiomatous tissue and an osteolytic pattern on X-rays, as well as negativity for hereditary, metabolic, neoplastic, immunologic and infectious etiologies.

In summary, although rare, the diagnosis of Gorham-Stout syndrome should be considered in cases of focal or multifocal osteolysis in previously healthy young individuals who test negative for inflammatory, infectious, metabolic, and neoplastic conditions.
